# Prevalence and associated factors of prehypertension in Afghanistan: a secondary data analysis of the 2018 STEPS survey

**DOI:** 10.3389/fpubh.2026.1705887

**Published:** 2026-03-19

**Authors:** Shah Jahan Shayan, Najma Siddiqi, Claire Carswell, Khalid Rehman, Obaidullah Fahim, Simon Walker, Zainab Samad, Ahmad Farshid Muhammadi, Gerardo A. Zavala

**Affiliations:** 1 Hull York Medical School, University of York, York, United Kingdom; 2 Department of Health Sciences, University of York, York, United Kingdom; 3 Bradford District Care NHS Foundation Trust, Bradford, United Kingdom; 4 Institute of Public Health and Social Sciences, Khyber Medical University, Peshawar, Pakistan; 5 Department of Nutrition, Faculty of Public Health, Kabul University of Medical Sciences “Abu Ali Ibn Sina”, Kabul, Afghanistan; 6 Centre for Health Economics, University of York, York, United Kingdom; 7 Department of Medicine, The Aga Khan University, Karachi, Pakistan; 8 Afghanistan National Institute of Public Health, Ministry of Public Health, Kabul, Afghanistan

**Keywords:** Afghanistan, associated factors, hypertension, prehypertension, prevalence

## Abstract

**Background:**

In Afghanistan, approximately 40% of adults aged 30–79 years are living with hypertension. The stage of prehypertension represents a critical point for early detection and prevention of hypertension and its complications. This study aims to determine the prevalence of prehypertension and explore its associations with socio-demographic, behavioural, and biological factors in the Afghan adult population.

**Methods:**

This study utilised data from the 2018 Afghanistan STEPS survey; a nationally representative, household-based, cross-sectional survey conducted from February to October 2018. Weighted estimates and multivariate logistic regression analyses were used to assess the prevalence of prehypertension and explore its associations with sociodemographic, behavioural, and biological factors.

**Results:**

The prevalence of prehypertension was 38.4%, and it was more prevalent among males (46.2%) than females (29.8%). Male sex (AOR: 2.66; 95% CI: 1.99–3.55) and high waist circumference (AOR: 2.14; 95% CI: 1.47–3.12) were found with higher odds of prehypertension. The prevalence of hypertension was 30.47%. Older age (45–69 years), obesity and high waist circumference associated with hypertension. Adults from the western and northeastern regions had lower odds of both prehypertension and hypertension.

**Conclusion:**

Prehypertension and hypertension are highly prevalent in Afghanistan. We identified high risk groups that could inform the development of interventions and modifiable risk factors which could be targeted in these interventions. Further research should focus on the development of contextualised, evidence-based prevention strategies.

## Introduction

Hypertension (HT) is a major global public health concern due to its high prevalence and association with cardiovascular diseases (CVDs) (1). According to the latest report from the Global Health Observatory, the prevalence of HT among adults aged 30–79, is 33.1% globally, with 34.5% of males and 31.7% of females having HT (2). In Afghanistan, the prevalence of HT in adults (30–79 years) is over 40%, exceeding global averages (2). Among Afghan males, the prevalence is 35.3%, while among females, it is 45.3% (2). As populations age, engage in unhealthy behaviors, and gain weight, the prevalence of HT continues to increase steadily (3).

The rising prevalence of HT is driven by modifiable and non-modifiable risk factors. Key modifiable behaviors include excessive salt intake (4), physical inactivity (5), tobacco use (6), alcohol consumption (7), and poor dietary habits (8). Which contribute to pathophysiological mechanisms including obesity, endothelial dysfunction, and arterial stiffness (9, 10). In addition to these behavioural factors, biological factors such as age, sex and genetic predisposition influence the development of HT. The interplay between these risk factors is particularly important in low and middle-income countries, where the epidemiological transition has led to a sharp rise in HT burden (11).

To enable earlier identification of individuals at risk of developing HT, the concept of prehypertension (PHT) was introduced by the Executive Committee of the Joint National Committee for Prevention, Detection, Evaluation, and Treatment of High Blood Pressure (JNC 7) (12). The aim was to identify people within the range of systolic blood pressure (SBP) between 120 and 139 mmHg and diastolic blood pressure (DBP) between 80 and 89 mmHg (12). Since HT is often asymptomatic and can lead to serious complications such as kidney failure, cardiovascular diseases (CVD) and stroke (13), introducing the concept of PHT made it easier to identify individuals who would benefit from early intervention through behavioural health risk modification and prevent its complications (14).

Longitudinal data from the Framingham Heart Study provided clear evidence that individuals with blood pressure (BP) levels higher than 120/80 had a higher risk of developing HT and cardiovascular disease (CVD) compared to those with BP levels below 120/80 mmHg (15). Additionally, a study conducted in Japan revealed that PHT was associated with a 45% higher risk of cardiovascular disease, even after adjusting for traditional cardiovascular disease risk factors (16). Moreover, individuals in the PHT range of BP were found to be 1.65 times more likely to have at least one additional risk factor compared to those with normal BP (16).

PHT prevalence varies globally. A cross-sectional study in China found a prevalence of 33.9% (17), while in Iran reported a prevalence of 28.5% (18). In India, Bangladesh and Nepal, the prevalence of PHT among adults aged 18–49 was reported 43.2, 35.1 and 25.2%, respectively (11). Age, obesity, smoking, family history of cardiovascular disease and waist circumference were reported as risk factors and determinants for PHT (17, 19).

In Afghanistan, traditionally, the public health agenda has been dominated by communicable diseases and conditions associated with maternal and child health (20). However, recent studies indicate that non-communicable diseases (NCDs) are rising and account for 50% of all deaths in the country, with HT identified as the leading risk factor (21). The situation is concerning given that the healthcare system is poorly equipped to manage chronic disease due to infrastructural deficiency, limited workforce capacity, and constrained financial resources at both system and household levels (22, 23). Moreover, access to NCDs-related care is predominantly centralised in tertiary level and private sector facilities, making essential services inaccessible for many, especially rural populations facing geographic and economic barriers (21). Following the political transition in 2021, women’s access to healthcare has declined further due to restricted policies on education, mobility and employment, increasing their exposure to NCDs risk factors such as psychological stress, inadequate physical inactivity and diet (24, 25). Simultaneously, the decline in international aid has exacerbated national economic collapse, reducing household purchasing power and altering dietary patterns in ways that contribute to both undernutrition and increased CVDs risk (23).

Despite the high burden of HT in Afghanistan, evidence on PHT remains sparse. Given the critical role of the PHT stage in preventing HT and its associated complications—particularly in resource-limited settings—this study seeks to address this gap by estimating its prevalence and associated factors in Afghanistan, using secondary analysis of the 2018 STEPS survey dataset. The findings have the potential to inform policy, guide public health strategies, and strengthen healthcare delivery approaches.

## Methods

### Study design and setting

This study used data from the 2018 nationally representative population-based and cross-sectional Afghanistan STEPS survey (Stepwise Approach to Surveillance) (26). This survey was conducted by the Ministry of Public Health of Afghanistan with the support of the World Health Organization (WHO). Its purpose was to determine the prevalence of non-communicable diseases and their associated risk factors in the country. Data was collected from February 2018 to October 2018.

### Sampling method and sample size

A multistage cluster sampling technique was used to obtain a nationally representative sample of adults (18–69 years). The sampling process included selecting 55 districts as primary sampling units, followed by villages or blocks as secondary sampling units, and households as tertiary sampling units. One person from each household was randomly selected, resulting in 3956 randomly selected participants (26).

### Data collection procedures and tools

Data collection was done in three steps. Step one involved administering a structured questionnaire to gather information on behavioural risk factors such as tobacco and alcohol use, physical activity, diet, and salt intake (26). Socio-demographic data, including age, sex, highest level of education, number of adult household members, and residence status, were also collected. Step two involved taking anthropometric measurements, including height and weight, using portable electronic weighing scales and inflexible measuring bars according to WHO protocols (26). Blood pressure was measured using a calibrated sphygmomanometer after participants had been seated for at least 15 min, with three readings (26). The last two blood pressure readings were averaged. Step three involved using CardioChek PA devices to measure blood glucose and cholesterol. The study forms were translated into Pashto and Dari and were piloted. Data collection was conducted through face-to-face interviews.

### Study variables

#### Outcome variables

PHT was defined according to the JNC7 as participants not taking antihypertensive drugs and having SBP 120–139 mmHg or DBP 80–89 mmHg (27). According to the JNC7, HT was defined as SBP ≥ 140 mmHg and/or DBP ≥ 90 mmHg, or self-reported use of antihypertensive medication (27). The prevalence of HT was also calculated based on the 2017 American College of Cardiology (ACC) and American Heart Association (AHA) guidelines, normal (<120/<80 mmHg), elevated (120–129/<80 mmHg), Stage 1 hypertension (130–139/80–89 mmHg), and Stage 2 hypertension (≥140/≥90 mmHg) (28).

#### Sociodemographic variables

We examined a range of sociodemographic characteristics: Sex (male, female); age group (18–29, 30–44, 45–69); residential area (rural, urban); region of the country (central, northern, southern, southeastern and eastern, western and northeastern); marital status (never married, married and widowed/separated); education level (no formal education, primary/secondary school, high school, university/college); work status (employed, unemployed, homemaker, student) and household earnings (divided into four quartiles).

#### Behavioural risk factors

For this study Current smoking (yes, no); ever alcohol drinking (yes, no); type of oil consumption (self-report type, vegetable oil, lard or suet, butter or ghee, margarine, non in particular); insufficient physical activity (based on WHO definition, less than 150 min of moderate-intensity physical activity or 75 min of vigorous-intensity physical activity in a week); low fruit and vegetable consumption (based on WHO recommendation, taking less than five servings of fruits and/or vegetables on average per day) (29); salt consumption (based on self-report amount, too much, right amount, too little, far too little); adding salt when eating (self-report, rarely, sometimes, often, always), were considered as possible behavioural risk factors.

#### Biological risk factors

BMI is categorised according to WHO guidelines as underweight <18.5, normal ≥18.5 to <25, overweight ≥25 to <30, obesity ≥30 kg/m^2^ (30). Central obesity, measured by waist circumference (WC) (based on WHO guidelines a waist circumference of >79 cm in females and >89 cm in males) (29). Fasting blood glucose levels classified by the American Diabetes Association as optimal <100, prediabetes ≥100 to <126, diabetes ≥126 mg/dL (31). Total cholesterol categorised as desirable <200, moderate ≥200 to <240, high ≥240 (49).

### Data management

The dataset was cleaned and weighted in survey mode to account for the complex survey design according to the World Health Organization’s recommendation (29). Participants with missing blood pressure measurements were excluded to ensure the accuracy of outcome classification. Additionally, biologically implausible blood pressure values (systolic blood pressure values less than 70 mmHg or greater than 250 mmHg, and diastolic blood pressure values less than 40 mmHg or greater than 150 mmHg) and age (individuals younger than 18 years or older than 69) were excluded to ensure data quality and reliability.

### Statistical analysis

Descriptive statistics were used to present the frequency distribution of sociodemographic characteristics of the participants. The weighted prevalence of blood pressure ranges was calculated by sex, age groups, residential area, regions of the country, and total population, along with 95% confidence intervals.

Logistic regression analyses were performed to examine the associations between selected factors and blood pressure categories. Two separate binary logistic regression models were developed: one comparing individuals with PHT to those with normal blood pressure (excluding those with HT) and another comparing individuals with HT to those with normal blood pressure (excluding those with PHT). Unadjusted odds ratios were estimated for each independent variable. Prior to model fitting, multicollinearity among independent variables was assessed.

To construct multivariable models, all variables with at least one category showing a statistically significant unadjusted association (*p*-value < 0.05) were initially considered. The “gvselect” command in STATA was used for best subset variable selection, evaluating all possible combinations of predictors. Model performance was assessed using Akaike Information Criterion (AIC) and Bayesian Information Criterion (BIC), and the model with the lowest values of AIC and BIC was selected. This approach was selected to identify the most parsimonious model that balanced model fit with complexity, thereby reducing the risk of overfitting and enhancing generalizability. An interaction term between gender and age group was included and tested using the Wald test. The final model’s fit was assessed using the F-adjusted goodness of fit.

As a sensitive analysis, an alternative logistic regression model was run in which individuals with either PHT or HT were grouped into a single “elevated blood pressure” category and compared to those with normal blood pressure. This approach tested the robustness of findings under an alternative outcome definition (32). The same model selection procedures, interaction testing, and goodness of fit assessments were applied.

All statistical analyses were performed using STATA version 18.0 (Stata Corporation, College Station, TX, USA) with a significance level set at *p* < 0.05.

## Result

### Characteristics of study population

**Figure 1:**
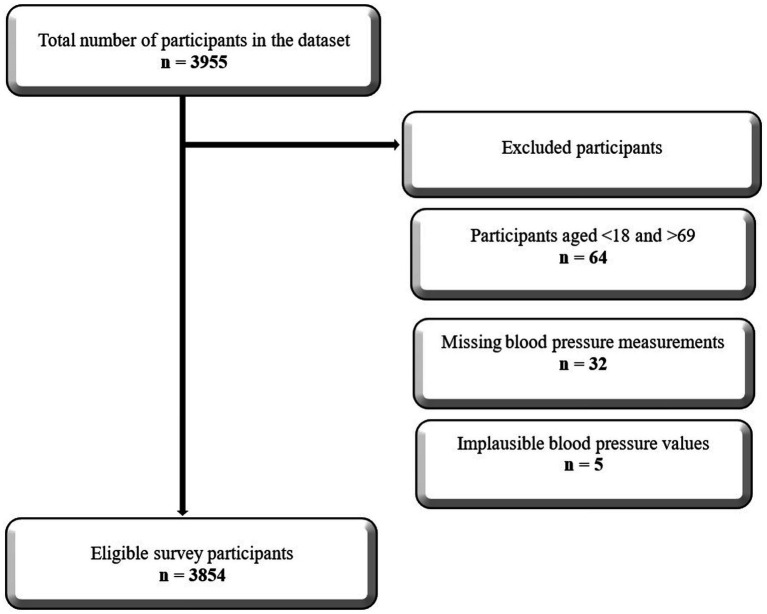
Participant exclusion flow diagram.

After excluding 101 participants under 18 and over 69 years of age, as well as outliers and cases with missing data on blood pressure (Appendix 1), 3,854 participants remained in the final analysis (Figure 1).

As presented in Table 1, over half of the sample were male (51.27%), and 37.34% were aged between 18 and 29 years. The majority of participants lived in urban areas (52.52%), and 80.72% of them were married. More than half of the participants (56.1%) had no formal education, and 45.8% were employed at the time of the survey.

**Table 1:** Characteristics of the survey participants.

Variables		Frequency (%)
Gender	Male	1,976 (51.27)
	Female	1,878 (48.73)
Age group`	18–29	1,439 (37.34)
	30–44	1,170 (30.36)
	45–69	1,245 (32.30)
Residential area	Rural	1,830 (47.48)
	Urban	2,024 (52.52)
Region of the country	Central	690 (17.90)
	Northern	662 (17.18)
	Southern	592 (15.36)
	Southeastern and eastern	656 (17.02)
	Western	623 (16.17)
	Northeastern	631 (16.37)
Marital status	Never married	582 (15.10)
	Married	3,111 (80.72)
	Widowed and divorced	161 (4.18)
Education level	No formal education	2,140 (56.08)
	Secondary/primary school	946 (24.79)
	High school	553 (14.49)
	College/university	177 (4.64)
Work status	Employed	1,761 (45.81)
	Unemployed	845 (21.98)
	Homemaker	1,105 (28.75)
	Student	133 (3.46)
Household earnings	Lowest quartile	848 (25.72)
	Second quartile	806 (24.45)
	Third quartile	874 (26.51)
	Highest quartile	769 (23.32)

### Weighted prevalence of PHT and HT

**Figure 2:**
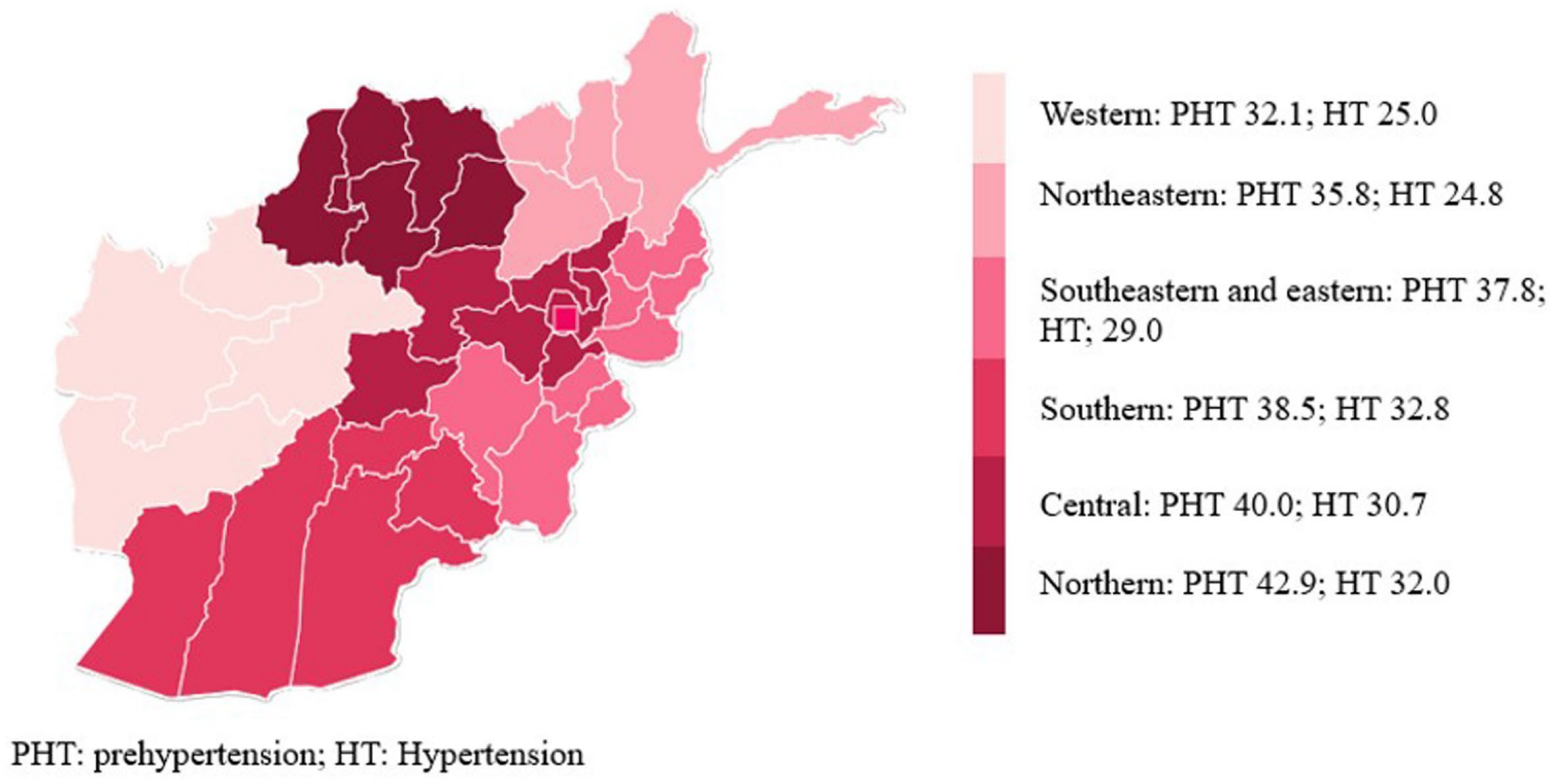
Prevalence of prehypertension and hypertension in six regions of Afghanistan.

Overall, 68.8% of the population had either PHT or HT (95% CI: 65.6–71.9%). In total, 38.4% of participants had PHT (95% CI: 35.1–41.7%), while 30.5% had HT (95% CI: 27.4–33.7%). As seen in Table 2, PHT was more common among males (46.2%; 95% CI: 42.4–50.1%) compared to females (29.8%; 95% CI: 26.3–33.6%), whereas HT was slightly more prevalent among females (32.7%; 95% CI: 29.3–36.3%) than males (28.3%; 95% CI: 24.1–33.0%). When stratified by age, PHT peaked in the 30-44-year age group (41.6%; 95% CI: 37.8–45.6), while HT peaked among adults aged 45–69 years (54.0; 95% CI: 47.0–60.9). Figure 2 shows the prevalence of PHT and HT in six regions of the country.

**Table 2:** Prevalence of PHT and HT by sociodemographic and biological characteristics of the participants.

Characteristics		*N*	Prevalence (95% CI)	
			Prehypertension	Hypertension
Gender	Male	1,976	46.27 (42.46–50.12)	28.37 (24.15–33.01)
	Female	1,878	29.89 (26.39–33.64)	32.74 (29.31–36.36)
Age group	18–29	1,439	40.83 (35.26–46.64)	20.93 (16.83–25.72)
	30–44	1,170	41.65 (37.84–45.56)	27.33 (22.19–33.14)
	45–69	1,245	28.92 (24.16–34.20)	54.02 (46.97–60.91)
Residential area	Rural	1,830	36.62 (32.74–40.68)	29.00 (24.06–34.50)
	Urban	2,024	39.71 (35.14–44.46)	31.53 (27.80–35.51)
BMI	Underweight	262	23.29 (14.52–35.18)	12.37 (6.52–22.20)
	Normal weight	1,734	44.66 (38.20–51.31)	22.00 (17.75–26.92)
	Overweight	1,054	38.68 (33.41–44.23)	42.69 (37.05–48.52)
	Obesity	629	31.57 (26.76–37.21)	54.99 (48.99–60.85)
WC	Normal WC	1,606	43.60 (39.24–48.06)	16.87 (13.89–20.33)
	High WC	2,076	35.88 (31.14–40.91)	43.41 (39.03–47.90)
Total			38.41 (35.16–41.77)	30.47 (27.41–33.70)

CI, confidence interval; BMI, body mass index; WC, waist circumference; high WC, >79 cm in females and >89 cm in males; underweight <18.5, normal weight ≥18.5 to <25, overweight ≥25 to <30, obesity ≥30 kg/m^2^.

Using the 2017 ACC and AHA blood pressure classification, 11.4% of participants were identified as having elevated blood pressure (95% CI: 9.1–14.3%), 26.9% had HT stage I (95% CI: 24.3–29.6%), and 30.4% had HT stage II (95% CI: 27.4–33.7%). According to this classification, the total prevalence of HT was 57.3% (95% CI: 53.1–61.5%).

### Factors associated with prehypertension

As shown in Table 3, males had significantly higher odds of PHT compared to females (AOR: 2.66; 95% CI: 1.99–3.55; *p* < 0.001). Additionally, participants with high waist circumference had significantly higher odds of PHT (AOR: 2.14; 95% CI: 1.47–3.12; *p* < 0.001) compared to those with normal waist circumference. Participants from the western region of the country had lower odds of PHT than those from the central region (AOR: 0.64; 95% CI: 0.42–0.98). The multicollinearity test indicated no significant associations between the independent variables. Results for the variables excluded during the multivariable model selection process, along with their unadjusted odds ratios and interaction test between age group and genderare presented in Appendices 2, 3.

**Table 3:** Multivariable logistic regression model for prehypertension.

Variables		Unadjusted odds (95% CI)	*p*-value	Adjusted odds (95% CI)	*p*-value
Gender	Female	Reference		Reference	
	Male	2.28 (1.62–3.20)	<0.001	2.66 (1.99–3.55)	<0.001
Age group	18–29	Reference		Reference	
	30–44	1.25 (0.97–1.63)	0.083	1.12 (0.86–1.46)	0.366
	45–69	1.58 (1.14–2.19)	0.006	1.38 (0.99–1.92)	0.052
Region of the country	Central	Reference		Reference	
	Northern	1.25 (0.84–1.86)	0.263	1.32 (0.80–2.19)	0.267
	Southern	0.98 (0.64–1.50)	0.940	1.19 (0.81–1.75)	0.357
	Southeastern and eastern	0.83 (0.56–1.23)	0.360	0.80 (0.51–1.28)	0.368
	Western	0.54 (0.37–0.79)	0.002	0.64 (0.42–0.98)	0.044
	Northeastern	0.66 (0.43–1.01)	0.061	0.71 (0.45–1.12)	0.144
WC	Normal	Reference		Reference	
	High	1.57 (1.08–2.26)	0.016	2.14 (1.47–3.12)	<0.001

CI, confidence interval; WC, waist circumference; high WC, >79 cm in females and >89 cm in males.

### Factors associated with hypertension

Participants aged 45–69 years had significantly higher odds of HT compared to those aged 18–29 in the adjusted model (AOR: 4.29; 95% CI: 2.44–7.51; *p* < 0.001). Regionally, participants from the northeastern part of the country had significantly lower odds of HT compared to those from the central region (AOR: 0.47; 95% CI: 0.28–0.79). Among the biological factors, obesity showed a strong and significant association with HT after adjustment (AOR: 3.03; 95% CI: 1.54–5.94; *p* < 0.001). Being underweight was associated with significantly lower odds of HT (AOR: 0.31; 95% CI: 0.12–0.81; *p* = 0.003), and high waist circumference was also associated with increased odds of HT (AOR: 2.58; 95% CI: 1.49–4.44; *p* = 0.001) (Table 4). Results for the variables excluded during the multivariable model selection process, along with their unadjusted odds ratios, are presented in Appendix 4.

**Table 4:** Multivariable logistic regression model for HT.

Variables		Unadjusted odds (95% CI)	*p*-value	Adjusted odds (95% CI)	*p*-value
Age group	18–29 years	Reference		Reference	
	30–44 years	1.60 (1.12–2.30)	0.009	1.32 (0.85–2.04)	0.207
	45–69 years	5.78 (3.60–9.27)	<0.001	4.29 (2.44–7.51)	<0.001
Region of the country	Central	Reference		Reference	
	Northern	1.21 (0.79–1.87)	0.365	1.18 (0.67–2.08)	0.553
	Southern	1.09 (0.74–1.60)	0.020	1.19 (0.72–1.95)	0.487
	Southeastern and eastern	0.83 (0.49–1.39)	0.489	0.68 (0.38–1.22)	0.198
	Western	0.55 (0.33–0.93)	0.026	0.62 (0.32–1.20)	0.158
	Northeastern	0.60 (0.36–0.98)	0.043	0.47 (0.28–0.79)	0.005
BMI	Normal	Reference		Reference	
	Overweight	3.47 (2.07–5.81)	<0.001	1.51 (0.69–3.29)	0.295
	Obesity	6.28 (3.65–10.81)	<0.001	3.03 (1.54–5.94)	<0.001
	Underweight	0.29 (0.11–0.71)	0.007	0.31 (0.12–0.81)	0.018
WC	Normal	Reference		Reference	
	High	4.91 (3.48–6.93)	<0.001	2.58 (1.49–4.44)	0.001
FBG	Optimal	Reference		Reference	
	Prediabetes	4.77 (2.65–8.60)	<0.001	1.67 (0.90–3.09)	0.101
	Diabetes	1.06 (0.68–1.65)	0.768	0.70 (0.19–2.53)	0.591
TC	Optimal	Reference		Reference	
	Moderate	2.38 (1.20–4.71)	0.013	1.65 (0.77–3.51)	0.189
	High	2.10 (0.87–5.06)	0.097	0.91 (0.24–3.44)	0.895

CI, confidence interval; BMI, body mass index; WC, waist circumference; FBG, fasting blood glucose; TC, total cholesterol; underweight <18.5, normal ≥18.5 to <25, overweight ≥25 to <30, obesity ≥ 30 kg/m^2^; high WC >79 cm in females and >89 cm in males; optimal FBG <100, prediabetes ≥100 to <126, diabetes ≥126 mg/dl; desirable TC <200, moderate TC ≥200 to <240, high TC ≥240.

All variables listed in the table were included in the multivariable to estimate adjusted odds ratios.

### Sensitivity analysis

Appendix 5 in the appendices presents an alternative multivariable logistic regression model where PHT and HT were combined into a single category (BP ≥ 120/80). In this model, being male was significantly associated with higher odds of PHT or HT (AOR: 2.29; 95% CI: 1.45–3.63; *p* = 0.001). Participants from the northeastern part of the country had lower odds of PHT or HT compared to those from the central region (AOR: 0.54; 95% CI: 0.35–0.83; *p* = 0.005). Widowed or divorced participants had significantly higher odds of PHT or HT than those who had never married (AOR: 6.02; 95% CI: 2.65–13.68; *p* < 0.001). Unemployment was also associated with higher odds of PHT or HT compared to employment (AOR: 1.83; 95% CI: 1.06–3.15; *p* = 0.028). High waist circumference (AOR: 2.03; 95% CI: 1.30–3.17; *p* = 0.002) and obesity (AOR: 2.95; 95% CI: 1.77–4.93; *p* < 0.001) were significantly associated with PHT or HT, while being underweight was associated with lower odds (AOR: 0.29; 95% CI: 0.12–0.66; *p* = 0.003).

## Discussion

To the best of the authors’ knowledge, this is the first study to examine the prevalence and associated factors of PHT in Afghanistan. We found that nearly seven in 10 Afghan adults (68.8%) aged 18–69 years lived either with PHT OR HT. The weighted prevalence of PHT and HT were 38.4 and 30.5%, respectively. Which is a critical public health problem, particularly given Afghanistan’s socio-economic and healthcare challenges (20). Higher odds of PHT were observed among males and individuals with high WC. For HT, the strongest association was with older age (45–69 years), followed by obesity and high WC, while adults from the western and northeastern regions had lower odds of both PHT and HT. In the combined model (BP ≥ 120/80 mmHg), being male, widowed/divorced, unemployed, obese, or having high WC were linked to higher odds of PHT or HT.

When compared with regional and international studies, the prevalence of PHT in Afghanistan (38.4%) is consistent with findings from other low- and middle-income countries (LMICs). For instance, studies from India and Bangladesh have reported PHT prevalence rates of 43.2 and 35.1% among adults, respectively (11). Similarly, a national survey in China reported a prevalence of 36.7% (33). Besides, our study estimated an HT prevalence of 30.47% in the country, slightly higher than the 27% regional average for South Asian Association for Cooperation (SAARC) countries, yet within the broad range of 13–47% reported across the region (34). This figure is also comparable to the global prevalence of approximately 32% (1) and mirrors findings from other LMICs undergoing rapid lifestyle and dietary changes (35).

The similarity of Afghanistan’s PHT and HT prevalence to that of other LMICs signals that the country is during the same epidemiological transition, from a predominance of communicable diseases to a rising burden of NCDs. However, unlike more economically stable LMICs, Afghanistan’s fragile health system, infrastructural deficiencies, and constrained financial resources leave it poorly prepared to address this shift (22). This combination of epidemiological transition and systemic fragility creates a scenario where the NCDs burden, particularly HT, is likely to grow unchecked unless urgent policy and health system interventions are implemented.

Higher odds of PHT were observed among males and individuals with high WC. These findings are consistent with previous studies that have highlighted gender (11) and central obesity (36) as important determinants of elevated blood pressure. Regarding HT, age was the factor with the highest estimate, with individuals aged 45–69 years having significantly greater odds of HT compared to those aged 18–29. This aligns with global evidence that age is the strongest non-modifiable risk factor due to vascular stiffening and accumulated exposure to other risk factors over time (11, 33, 37). Similarly, obesity and high WC were also consistently associated with HT, echoing findings from multiple LMIC settings, where central obesity has been strongly linked to increased vascular resistance and higher BP levels (36, 38, 39).

Adults in the western and northeastern regions had significantly lower odds of PHT and HT, respectively, compared to those from the central region. This could reflect differences in dietary patterns, socioeconomic status, healthcare access, or mental health status. Similar intra-country geographic variations have been reported in south Asian countries, emphasizing the role of local contextual factors (11). Mental health appears to be a critical yet underexplored factor in understanding PHT and HT patterns in Afghanistan. A recent national survey reported alarmingly high prevalence rates of depression (72.05%), anxiety (71.94%), and stress (66.49%) among Afghan adults (40). Multivariable analysis from the same study identified female gender, low economic status, rural residency, illiteracy, cigarette smoking, and recent exposure to adverse life events as significant predictors of poor mental health outcomes (40). Notably, a meta-analysis has shown that depression increases the risk of developing HT by approximately 1.5 times and may represent an independent risk factor (41). Such evidence suggests that the high psychosocial stress burden in Afghanistan could contribute substantially to the overall prevalence of both PHT and HT, help explain the higher rates observed among women and account for part of the observed regional variation in odds ratios.

The study also highlighted the protective effects of being underweight against HT. While undernutrition is generally undesirable, several studies have observed inverse associations between low BMI and HT (42, 43). However, the health risks associated with underweight status should not be overlooked.

For the combined category of BP ≥ 120/80 mmHg, additional significant associations included being widowed or divorced and unemployment with having higher odds of elevated blood pressure, both of which may reflect the psychosocial stress pathway. These findings suggest that social determinants of health, such as marital status and employment, can contribute to blood pressure elevation through stress-related mechanisms and reduced health-seeking behaviors (44).

A pattern was observed between the prevalence of PHT and HT across age and BMI categories. Younger adults aged 18–29 had the highest prevalence of PHT (40.8%) but the lowest prevalence of HT (20.9%), whereas the oldest age group (45–69 years) had the lowest PHT (28.9%) and highest HT (54.0%) prevalence. A similar pattern was seen in BMI categories, where normal-weight individuals had the highest PHT (44.6%) but lower HT (22.0%), while obese individuals had a lower PHT rate (31.5%) and significantly higher HT (54.9%). This trend reflects the natural progression of BP from normal to PHT, and eventually to HT, emphasising the need for early identification and intervention during the PHT stage, particularly among younger and normal-weight individuals.

In Afghanistan, given the challenging situation in terms of socio-economic, health system capacity and literacy level, implementing culturally appropriate, low-cost prevention strategies could be particularly effective. Modifying weight status through dietary approaches, increasing awareness about WC and promoting physical activity can be key components. Educational campaigns tailored to different literacy levels and regional practices could enhance effectiveness.

One of the cost-effective strategies to manage NCDs is self-management, which could be viable to the given context (45). Evidence from randomised controlled trials and systematic reviews suggests that self-management approaches, including dietary modification, physical activity, self-monitoring, and health education, can significantly reduce blood pressure levels (27, 46, 47). For example, the PREMIER trial in the USA demonstrated that lifestyle changes, including reduced sodium intake and increased physical activity, resulted in significant reductions in systolic and diastolic BP among prehypertensive adults (48).

### Strengths and limitations

This study used Afghanistan’s 2018 STEPS survey data, which provided standardized, internationally comparable NCDs associated factor measurements through its multistage cluster sampling design, offering the first nationally representative assessment of Afghanistan’s NCDs burden. The inclusion of behavioural, clinical, and biochemical measurements enables comprehensive risk factor profiling. However, limitations include potential self-report biases in behavioural data, reduced current applicability due to post-2018 sociopolitical changes (particularly regarding women’s health access), and the inability to establish causality inherent to cross-sectional designs. Important unmeasured or incompletely measured confounders include mental health indicators, socioeconomic factors, detailed dietary intake, environmental exposure and cultural determinants of health behaviors. Future research should employ longitudinal designs with biomarker validation and incorporate Afghanistan-specific social determinants to address these gaps while accounting for the country’s evolving health landscape.

The standardized measures that have been widely validated across STEPS surveys globally for population-level surveillance, minimise the issues. Besides, the design enhances comparability with international findings. Despite its limitations, this study delivers essential evidence to inform future research and public health interventions.

## Conclusion

This study reveals a high burden of PHT and HT in Afghanistan, with significant associations observed with male sex, high WC and geographic region. The identified associated factors highlight high risk groups and can inform interventions. Notably, some of these factors are modifiable, offering valuable opportunities for early prevention of hypertension. However, further longitudinal studies are needed to establish causal relationships and guide the development of effective, evidence-based prevention strategies.

## Data Availability

The datasets presented in this article are not readily available because the dataset used in this study was obtained from the World Health Organization (WHO) 2018 STEPS survey conducted in Afghanistan. Access to the dataset is subject to WHO data sharing policies and restrictions. Researchers may request access directly from the WHO by submitting a formal application through the WHO NCD Microdata Repository. The authors are not permitted to share the dataset publicly. Requests to access the datasets should be directed to https://extranet.who.int/ncdsmicrodata.

## References

[1] Zhou B Carrillo-Larco RM Danaei G Riley LM. Worldwide trends in hypertension prevalence and progress in treatment and control from 1990 to 2019: a pooled analysis of 1201 population-representative. Lancet. (2021) 398:957–80. 34450083 10.1016/S0140-6736(21)01330-1 PMC8446938

[2] World Health Organization. Age-Standardized Prevalence of Hypertension Among Adults Aged 30–79 Years (%). Geneva: World Health Organization (2019).

[3] Lip GYH Coca A Kahan T Boriani G Manolis AS Olsen MH . Hypertension and cardiac arrhythmias: executive summary of a consensus document from the European heart rhythm association (EHRA) and ESC Council on hypertension, endorsed by the Heart Rhythm Society (HRS), Asia-Pacific Heart Rhythm Society (APHRS), and Sociedad Latinoamericana de Estimulación Cardíaca y Electrofisiología (SOLEACE). Eur Heart J Cardiovasc Pharmacother. (2017) 3:235–50. doi: 10.1093/ehjcvp/pvx019, 28541499 PMC13588646

[4] He FJ Tan M Ma Y MacGregor GA. Salt reduction to prevent hypertension and cardiovascular disease: JACC state-of-the-art review. J Am Coll Cardiol. (2020) 75:632–47. doi: 10.1016/j.jacc.2019.11.055, 32057379

[5] Wang C Zeng J Liu H Zhang L. Causal relationship between physical activity, sleep duration, sedentary behavior, and hypertension: a Mendelian randomisation study. Am J Prev Cardiol. (2025) 22:101005. doi: 10.1016/j.ajpc.2025.101005 40487248 PMC12143605

[6] Yamato I Kansui Y Matsumura K Inoue M Ibaraki A Sakata S . Impact of smoking status on incident hypertension in a Japanese occupational population. Hypertens Res. (2025) 48:180–8. doi: 10.1038/s41440-024-01996-x, 39516368 PMC11832419

[7] Cecchini M Filippini T Whelton PK Iamandii I Di Federico S Boriani G . Alcohol intake and risk of hypertension: a systematic review and dose-response meta-analysis of nonexperimental cohort studies. Hypertension. (2024) 81:1701–15. doi: 10.1161/HYPERTENSIONAHA.124.22703, 38864208 PMC11251509

[8] Zhao Q Wu Q Zhong H Yan B Wu J Guo W. Association of dietary habits with body mass index and waist circumference, and their interaction effect on hypertension. Medicine (Baltimore). (2024) 103:e38178. doi: 10.1097/MD.0000000000038178, 38758876 PMC11098196

[9] Clayton TL Fitch A Bays HE. Obesity and hypertension: obesity medicine association (OMA) clinical practice statement (CPS) 2023. Obes Pillars. (2023) 8:100083. doi: 10.1016/j.obpill.2023.100083 38125655 PMC10728712

[10] Wu S Song L Wang L Chen S Wu M Wang Y . Transitions in metabolic health and associations with arterial stiffness progression across body mass index categories. Hypertension. (2021) 78:1270–7. doi: 10.1161/HYPERTENSIONAHA.121.17735, 34488437

[11] Rahut DB Mishra R Sonobe T Timilsina RR. Prevalence of prehypertension and hypertension among the adults in South Asia: a multinomial logit model. Front Public Health. (2022) 10:1006457. doi: 10.3389/fpubh.2022.1006457, 36777775 PMC9911430

[12] Lenfant C Chobanian AV Jones DW Roccella EJ. Seventh report of the joint national committee on the prevention, detection, evaluation, and treatment of high blood pressure (JNC 7). Circulation. (2003) 107:2993–4. doi: 10.1161/01.cir.0000080481.62058.03, 12756222

[13] Sanusi A. "Consequences of hypertension". In: Kibel DA, editor. Blood Pressure Physiology [Working Title]. London: IntechOpen (2025). doi: 10.3389/fpubh.2022.1006457

[14] Li W Liu H Wang X Liu J Xiao H Wang C . Interventions for reducing blood pressure in prehypertension: a meta-analysis. Front Public Health. (2023) 11:1139617. doi: 10.3389/fpubh.2023.1139617, 37033077 PMC10078829

[15] Vasan RS Larson MG Leip EP. Impact of high-normal blood pressure on the risk of cardiovascular disease. ACC Curr J Rev. (2002) 11:31. doi: 10.1016/s1062-1458(02)00536-6 11794147

[16] Ishikawa Y Ishikawa J Ishikawa S Kario K Kajii E. Jichi medical school cohort investigators group. Progression from prehypertension to hypertension and risk of cardiovascular disease. J Epidemiol. (2017) 27:8–13. doi: 10.1016/j.je.2016.08.001, 28135198 PMC5328734

[17] Song J Chen X Zhao Y Mi J Wu X Gao H. Risk factors for prehypertension and their interactive effect: a cross- sectional survey in China. BMC Cardiovasc Disord. (2018) 18:182. doi: 10.1186/s12872-018-0917-y, 30219041 PMC6139180

[18] Najafipour H Nasri HR Rostamzadeh F Amirzadeh R Shadkam M Mirzazadeh A. Prevalence and incidence of pre-hypertension and hypertension (awareness/control) in Iran: findings from Kerman coronary artery diseases risk factors study 2 (KERCADRS). J Hum Hypertens. (2022) 36:461–72. doi: 10.1038/s41371-020-00392-5, 32929131

[19] Jang I. Pre-hypertension and its determinants in healthy young adults: analysis of data from the Korean National Health and nutrition examination survey VII. Int J Environ Res Public Health. (2021) 18:9144. doi: 10.3390/ijerph18179144, 34501734 PMC8431073

[20] Saeed KMI. Prevalence of risk factors for non-communicable diseases in the adult population of urban areas in Kabul City, Afghanistan. Cent Asian J Glob Health. (2013) 2:69. doi: 10.5195/cajgh.2013.69, 29755883 PMC5927744

[21] Neyazi N Mosadeghrad AM Tajvar M Safi N. Trend analysis of noncommunicable diseases and their risk factors in Afghanistan. Chronic Dis Transl Med. (2023) 9:210–21. doi: 10.1002/cdt3.62, 37711869 PMC10497825

[22] Mirzazada S Padhani ZA Jabeen S Fatima M Rizvi A Ansari U . Impact of conflict on maternal and child health service delivery: a country case study of Afghanistan. Confl Heal. (2020) 14:38. doi: 10.1186/s13031-020-00285-x, 32536966 PMC7288441

[23] World Bank. Afghanistan Development Update Navigating Challenges: Confronting Economic Recession and Deflation [Internet]. Washington, D.C.: World Bank (2024).

[24] Qaderi S Miranda AV Odey GO Musa SS Sy Lim LT Vicente CR . Taliban’s war on educating girls and women must end now: a call for global actions. Public Health Challenges. (2023) 2:e80. doi: 10.1002/puh2.80, 40495866 PMC12039753

[25] Tawfiq E Azimi MD Feroz A Hadad AS Soroush MS Jafari M . Predicting maternal healthcare seeking behaviour in Afghanistan: exploring sociodemographic factors and women’s knowledge of severity of illness. BMC Pregnancy Childbirth. (2023) 23:561. doi: 10.1186/s12884-023-05750-y, 37533023 PMC10398983

[26] World Health Organization. STEPS 2018, Afghanistan. Geneva: World Health Organization (2020).

[27] Chobanian AV Bakris GL Black HR. The seventh report of the joint National Committee on prevention, detection, evaluation, and treatment of high blood pressure. The JNC 7 report. ACC Curr J Rev. (2003) 12:31–2. doi: 10.1016/S1062-1458(03)00270-8 14656957

[28] Whelton PK Carey RM Aronow WS Casey DE Jr Collins KJ Dennison Himmelfarb C . 2017 ACC/AHA/AAPA/ABC/ACPM/AGS/APhA/ASH/ASPC/NMA/PCNA guideline for the prevention, detection, evaluation, and management of high blood pressure in adults: a report of the American college of cardiology/American heart association task force on clinical practice guidelines. J Am Coll Cardiol. (2018) 71:e127–248. doi: 10.1016/j.jacc.2017.11.006, 29146535

[29] World Health Organisation. Noncommunicable Disease Surveillance, Monitoring and Reporting. Geneva: World Health Organisation (n.d.).

[30] Consultation WHO. Obesity: preventing and managing the global epidemic. Report of a WHO consultation. World Health Organ Tech Rep Ser. (2000) 894:1–253. 11234459

[31] American Diabetes Association Professional Practice Committee. 2. Diagnosis and classification of diabetes: standards of care in diabetes-2024. Diabetes Care. (2024) 47:S20–42. doi: 10.2337/dc24-S002 38078589 PMC10725812

[32] Saltelli A. Sensitivity analysis for importance assessment. Risk Anal. (2002) 22:579–90. doi: 10.1111/0272-4332.00040, 12088235

[33] Shen Y Chang C Zhang J Jiang Y Ni B Wang Y. Prevalence and risk factors associated with hypertension and prehypertension in a working population at high altitude in China: a cross-sectional study. Environ Health Prev Med. (2017) 22:19. doi: 10.1186/s12199-017-0634-7, 29165123 PMC5664790

[34] Neupane D Mclachlan C Sharma R Gyawali B Khanal V Mishra S . Prevalence of hypertension in member countries of south Asian Association for Regional Cooperation (SAARC): systematic review and meta-analysis. Medicine (Baltimore). (2014) 93:e74. doi: 10.1097/MD.0000000000000074 25233326 PMC4616265

[35] Ibrahim MM Damasceno A. Hypertension in developing countries. Lancet. (2012) 380:611–9. doi: 10.1016/S0140-6736(12)60861-7, 22883510

[36] Deng W-W Wang J Liu M-M Wang D Zhao Y Liu Y-Q . Body mass index compared with abdominal obesity indicators in relation to prehypertension and hypertension in adults: the CHPSNE study. Am J Hypertens. (2013) 26:58–67. doi: 10.1093/ajh/hps001, 23382328

[37] Lin Y Lai X Chen G Xu Y Huang B Chen Z . Prevalence and risk factors associated with prehypertension and hypertension in the Chinese she population. Kidney Blood Press Res. (2012) 35:305–13. doi: 10.1159/000336085, 22377586

[38] Nurdiantami Y Watanabe K Tanaka E Pradono J Anme T. Association of general and central obesity with hypertension. Clin Nutr. (2018) 37:1259–63. doi: 10.1016/j.clnu.2017.05.012, 28583324

[39] Seenappa K Kulothungan V Mohan R Mathur P. District-wise heterogeneity in blood pressure measurements, prehypertension, raised blood pressure, and their determinants among Indians: National Family Health Survey-5. Int J Public Health. (2024) 69:1606766. doi: 10.3389/ijph.2024.1606766, 38562553 PMC10982880

[40] Neyazi A Mohammadi AQ Razaqi N Rahimi BA Sifat S Rahimy N . Health survey on anxiety, depression, and stress in Afghanistan: a large-scale cross-sectional study amid ongoing challenges. Discov Ment Health. (2024) 4:38. doi: 10.1007/s44192-024-00090-5, 39302527 PMC11415558

[41] Meng L Chen D Yang Y Zheng Y Hui R. Depression increases the risk of hypertension incidence: a meta-analysis of prospective cohort studies. J Hypertens. (2012) 30:842–51. doi: 10.1097/HJH.0b013e32835080b7, 22343537

[42] Ghosh S Mukhopadhyay S Barik A. Sex differences in the risk profile of hypertension: a cross-sectional study. BMJ Open. (2016) 6:e010085. doi: 10.1136/bmjopen-2015-010085 PMC4964242 27466234

[43] Mosha NR Mahande M Juma A Mboya I Peck R Urassa M . Prevalence,awareness and factors associated with hypertension in north West Tanzania. Glob Health Action. (2017) 10:1321279. doi: 10.1080/16549716.2017.1321279, 28598724 PMC5496079

[44] Liu M-Y Li N Li WA Khan H. Association between psychosocial stress and hypertension: a systematic review and meta-analysis. Neurol Res. (2017) 39:573–80. doi: 10.1080/01616412.2017.1317904, 28415916

[45] Ahn S Basu R Smith ML Jiang L Lorig K Whitelaw N . The impact of chronic disease self-management programs: healthcare savings through a community-based intervention. BMC Public Health. (2013) 13:1141. doi: 10.1186/1471-2458-13-1141, 24314032 PMC3878965

[46] Li R Liang N Bu F Hesketh T. The effectiveness of self-management of hypertension in adults using mobile health: systematic review and meta-analysis. JMIR Mhealth Uhealth. (2020) 8:e17776. doi: 10.2196/17776, 32217503 PMC7148553

[47] Hany A Putra KR Vatmasari RA Nafis AN Amalia AT Khamdani E. Importance of self-management interventions in hypertension patients: a scoping review. Healthcare Low-Resource Sett. (2024). 12(s1):13434. doi: 10.4081/hls.2024.13034

[48] PREMIER Collaborative Research Group: Appel LJ Champagne CM Harsha DW Cooper LS Obarzanek E Elmer PJ . Effects of comprehensive lifestyle modification on blood pressure control. JAMA. (2003) 289:2083–93. doi: 10.1001/jama.289.16.2083, 12709466

[49] Grundy SM Stone NJ Bailey AL Beam C Birtcher KK Blumenthal RS . “2018 AHA/ACC/AACVPR/AAPA/ABC/ACPM/ADA/AGS/APhA/ASPC/NLA/PCNA Guideline on the Management of Blood Cholesterol: A Report of the American College of Cardiology/American Heart Association Task Force on Clinical Practice Guidelines: A Report of the American College of Cardiology/American Heart Association Task Force on Clinical Practice Guidelines”. Circulation. (2019) 139:e1082–1143., 30586774 10.1161/CIR.0000000000000625 PMC7403606

