# Supplementary material for: Prevalence and associated factors of prehypertension in Afghanistan: a secondary data analysis of the 2018 STEPS survey
Source: Front Public Health. 2026 Mar 19;14:1705887. doi: 10.3389/fpubh.2026.1705887 (PMC13044068; doi:10.3389/fpubh.2026.1705887)
Supplement: Supplementary file 1 [file Table_1.docx]

**Appendix 1.** Sociodemographic distribution of participants with missing blood pressure measurement data

| **Sociodemographic characteristics** | | **Number of participants** |
| --- | --- | --- |
| **Gender** | Male | 13 |
|  | Female | 19 |
| **Age group** | 18-29 | 24 |
|  | 30-44 | 6 |
|  | 44-69 | 2 |
| **Region of the country** | Northern | 4 |
|  | Northeastern | 3 |
|  | Western | 9 |
|  | Central | 5 |
|  | Southern | 2 |
|  | Southeastern and East | 9 |
| **Total** | | **32** |

**Appendix 2.** Variables excluded during multivariate model selection: Unadjusted odds for prehypertension

| **Variables (reference)** | | **Unadjusted odds (95% CI)** | **P-value** |
| --- | --- | --- | --- |
| Residential area (rural) | Urban | 1.29 (0.97 – 1.72) | 0.076 |
| Marital status (never married) | Married | 0.95 (0.68 – 1.32) | 0.762 |
|  | Widowed/divorced | 1.38 (0.62 – 3.05) | 0.626 |
| Education level (no formal education) | Primary/secondary school | 1.29 (0.97 – 1.71) | 0.072 |
|  | High school | 1.13 (0.54 – 2.36) | 0.738 |
|  | University/college | 0.96 (0.42 – 2.18) | 0.923 |
| Work status (employed) | Unemployed | 0.83 (0.50 – 1.39) | 0.492 |
|  | Homemaker | 0.46 (0.31 – 0.67) | <0.001 |
|  | Student | 1.43 (0.66 – 3.13) | 0.358 |
| Household earnings (first quartile) | Second quartile | 1.20 (0.77 – 1.87) | 0.404 |
|  | Third quartile | 0.78 (0.52 – 1.18) | 0.244 |
|  | Highest quartile | 1.32 (0.81 – 2.14) | 0.262 |
| Current smoking (no) | Yes | 1.39 (0.94 – 2.05) | 0.091 |
| Smokeless tobacco (no) | Yes | 1.80 (1.12 – 2.89) | 0.015 |
| Ever drinking alcohol (no) | Yes | 1.06 (0.41 – 2.77) | 0.895 |
| Type of oil consumption (vegetable oil) | Lard of suet | 0.32 (0.14 – 0.74) | 0.008 |
|  | Butter or ghee | 1.01 (0.73 – 1.39) | 0.932 |
|  | Margarine | 4.39 (0.50 - 38.29) | 0.179 |
|  | Non in particular | 1.08 (0.57 – 2.05) | 0.807 |
| Amount of salt consumption (right amount) | Far too much | 0.81 (0.45 – 1.44) | 0.478 |
|  | Too much | 1.39 (0.76 – 2.54) | 0.270 |
|  | Too little | 0.67 (0.37 – 1.22) | 0.197 |
|  | Far too little | 0.58 (0.21 – 1.54) | 0.278 |
| Adding salt when eating (never) | Rarely | 1.30 (0.90 – 1.89) | 0.156 |
|  | Sometimes | 1.18 (0.83 – 1.69) | 0.337 |
|  | Often | 1.44 (0.94 – 2.22) | 0.090 |
|  | Always | 1.13 (0.75 – 1.70) | 0.550 |
| Physical activity (sufficient) | Insufficient | 0.95 (0.50 – 1.82) | 0.871 |
| Vegs/fruits (adequate) | Inadequate | 1.11 (0.80 – 1.55) | 0.505 |
| BMI (normal) | Overweight | 1.54 (0.93 – 2.56) | 0.089 |
|  | Obesity | 1.78 (1.05 – 3.02) | 0.030 |
|  | Underweight | 0.27 (0.11 – 0.65) | 0.004 |
| FBG (optimal) | Prediabetes | 1.24 (0.76 – 2.01) | 0.380 |
|  | Diabetes | 1.14 (0.72 – 1.79) | 0.572 |
| TC (optimal) | Moderate | 0.78 (0.38 – 1.61) | 0.511 |
|  | High | 1.00 (0.39 – 2.54) | 0.989 |

**Appendix 3.** Joint test for interaction between gender and age group

| **Model outcome** | **Interaction tested** | **Adjusted Wald test (F, df)** | **p-value** |
| --- | --- | --- | --- |
| Prehypertension | gender × age group | F (2,264) = 1.33 | 0.266 |

**Appendix 4.** Variables excluded during multivariate model selection: Unadjusted odds for hypertension

| **Variables (reference)** | | **Unadjusted odds (95% CI)** | **P-value** |
| --- | --- | --- | --- |
| Gender (female) | Male | 1.27 (0.89 – 1.82) | 0.176 |
| Residential area (rural) | Urban | 1.29 (0.92 – 1.83) | 0.135 |
| Marital status (never married) | Married | 2.46 (1.61 – 3.78) | <0.001 |
|  | Widowed/divorced | 13.80 (6.44 – 29.58) | <0.001 |
| Education level (no formal education) | Primary/secondary school | 0.91 (0.68 – 1.23) | 0.564 |
|  | High school | 0.59 (0.28 – 1.23) | 0.161 |
|  | University/college | 0.32 (0.11 – 0.90) | 0.031 |
| Work status (employed) | Unemployed | 1.25 (0.69 – 2.28) | 0.449 |
|  | Homemaker | 0.85 (0.57 – 1.25) | 0.418 |
|  | Student | 0.64 (0.16 – 2.56) | 0.535 |
| Household earnings (first quartile) | Second quartile | 1.34 (0.91 – 1.97) | 0.125 |
|  | Third quartile | 0.94 (0.55 – 1.58) | 0.817 |
|  | Highest quartile | 1.48 (0.85 – 2.59) | 0.163 |
| Current smoking (no) | Yes | 1.00 (0.47 – 2.12) | 0.990 |
| Smokeless tobacco (no) | Yes | 2.26 (1.47 – 3.49) | <0.001 |
| Ever drinking alcohol (no) | Yes | 4.32 (1.29 – 14.04) | 0.017 |
| Type of oil consumption (vegetable oil) | Lard of suet | 0.16 (0.03 – 0.78) | 0.024 |
|  | Butter or ghee | 1.00 (0.73 – 1.35) | 0.987 |
|  | Margarine | 1.14 (0.32 – 4.03) | 0.837 |
|  | Non in particular | 1.29 (0.58 – 2.83) | 0.523 |
| Amount of salt consumption (right amount) | Far too much | 0.84 (0.38 – 1.84) | 0.669 |
|  | Too much | 1.50 (0.90 – 2.48) | 0.114 |
|  | Too little | 1.46 (0.94 – 2.28) | 0.087 |
|  | Far too little | 1.97 (0.88 – 4.41) | 0.095 |
| Adding salt when eating (never) | Rarely | 1.48 (0.82 – 2.68) | 0.186 |
|  | Sometimes | 0.73 (0.50 – 1.09) | 0.131 |
|  | Often | 0.67 (0.39 – 1.15) | 0.153 |
|  | Always | 0.63 (0.37 – 1.06) | 0.087 |
| Physical activity (sufficient) | Insufficient | 0.84 (0.42 – 1.69) | 0.634 |
| Vegs/fruits (adequate) | Inadequate | 1.28 (0.75 – 2.17) | 0.355 |

**Appendix 5.** Multivariate logistic regression model for PHT or HT (BP≥120/80)

| **Variables (reference)** | | **Adjusted odds (95% CI)** | **P-value** |
| --- | --- | --- | --- |
| Gender (female) | Male | 2.29 (1.45 – 3.63) | <0.001 |
| Region of the country (central) | Northern | 1.28 (0.85 – 1.93) | 0.223 |
|  | Southern | 1.14 (0.79 – 1.65) | 0.458 |
|  | Southeastern and eastern | 0.67 (0.41 – 1.09) | 0.112 |
|  | Western | 0.74 (0.47 – 1.16) | 0.192 |
|  | Northeastern | 0.54 (0.35 – 0.83) | 0.005 |
| Marital status (never married) | Married | 1.54 (0.94 – 2.52) | 0.080 |
|  | Widowed and divorced | 6.02 (2.65 – 13.68) | <0.001 |
| Work status (employed) | Unemployed | 1.83 (1.06 – 3.15) | 0.028 |
|  | Homemaker | 0.95 (0.53 – 1.72) | 0.890 |
|  | Student | 2.73 (0.93 – 7.99) | 0.066 |
| Waist circumference (normal) | High | 2.03 (1.30 – 3.17) | 0.002 |
| BMI (normal) | Overweight | 1.62 (0.92 – 2.84) | 0.092 |
|  | Obesity | 2.95 (1.77 – 4.93) | <0.001 |
|  | Underweight | 0.29 (0.12 – 0.66) | 0.003 |
